# Immunoglobulin substitution in patients with secondary antibody deficiency in chronic lymphocytic leukemia and multiple myeloma: a representative analysis of guideline adherence and infections

**DOI:** 10.1007/s00520-022-06920-y

**Published:** 2022-03-07

**Authors:** Hartmut Link, Markus Kerkmann, Laura Holtmann, for the Working Groups Supportive Care (AGSMO), Medical Oncology (AIO) of the German Cancer Society (DKG)

**Affiliations:** 1 Internal Medicine, Hematology and Medical Oncology, Kaiserslautern, Finkenhain 8, D-67661 Kaiserslautern, Germany; 2 MMF GmbH, Lindberghweg 132, D-48155 Münster, Germany

**Keywords:** Immunoglobulin replacement therapy, Secondary IgG deficiency, Guidelines, Infections comorbidity, Hematological malignancies

## Abstract

**Introduction:**

In secondary immunodeficiency, immunoglobulin replacement therapy (IgRT) is recommended by guidelines (GL) for patients with IgG level < 4 g/l and more than 3 infections or a severe infection. IgRT may be appropriate if IgG level < 4 g/l and/or 1–3 less severe infections (≤ grade 2).

**Methods:**

This was a retrospective sample analysis representative for practices and hospitals in Germany. The treatments and infection data were collected from patients with chronic lymphocytic leukemia (CLL) and multiple myeloma (MM). GL adherence (GLAD) was analyzed.

**Results:**

Data from 1086 patients (CLL 490, MM 596) were collected from 86 centers. Of all patients, 34.8% developed IgG deficiency during therapy (CLL 35.5%; MM 34.2%). IgRT was given in 23.5% of CLL and 14.4% of MM patients. GLAD in hypogammaglobulinemia and indication to IgRT was 23.3% of 86 CLL and 22.1% of 77 MM patients. Without GLAD, the hazard ratio (HR) for any infection was 4.49 (95% CI 3.72–5.42; *p* < 0.001) and for severe infections (grade ≥ 3) 10.64 (95% CI 7.54–15.00; *p* < 0.001). Significant independent risk factors for infections were a higher Charlson Comorbidity Index, IgG deficiency, and 3^rd^ + line treatment, as well as therapy with BTK inhibitors or chemotherapy in CLL. Multivariable analysis showed a significantly lower risk of severe infections after start of IgRT with a HR of 0.47 (95% CI 0.28–0.77; *p* = 0.003).

**Conclusions:**

Guideline adherence correlated with fewer and less severe infections but was low in patients with indication to IgRT. Risk factors for infection can be identified. Risk of severe infections was significantly lower in patients with IgRT.

**Supplementary Information:**

The online version contains supplementary material available at 10.1007/s00520-022-06920-y.

Infections are a common cause of death in patients with CLL and MM [1–3]. In addition, drug therapy for malignant diseases can exacerbate or cause immunodeficiency, lymphocytopenia, and neutropenia [4–6], increasing the risk of bacterial, viral, and fungal infections, also those caused by opportunistic pathogens [7, 8]. Infections account for the greatest proportion of deceasing CLL patients [9]. In case of a deficiency of class G immunoglobulins (IgG), which is disease associated in patients with CLL and MM [4], infections occur more frequently. Regular substitution therapy with IgG (IgRT) has become the standard of care for secondary antibody deficiency and frequent infections in order to reduce the rate of bacterial infections in CLL and MM [10–13].

Therefore, guidelines from professional societies [14, 15], the European Medicines Agency (EMA) [16], the German Medical Association (BÄK) [17], and the German Society for Hematology and Medical Oncology (DGHO) [15] recommend IgG substitution for secondary immunodeficiencies (SID). SID are defined as hypogammaglobulinemia with additional clinical manifestations: patients suffering from severe or recurrent infections, ineffective antimicrobial treatment, and either proven specific antibody failure (PSAF) [16]. The recommended dose for IgG substitution is in these cases is 0.2–0.4 g/kg body weight every 3 to 4 weeks.

The disease-specific guidelines for CLL and MM recommend IgRT under similar conditions [18, 19]. This study investigated the quality of guideline adherence (GLAD) in existing indications for IgG substitution in patients with CLL and MM in Germany.

## Methods

Patients from practices and hospitals in Germany who were representatively screened using previously collected care parameters of the participating institutions were analyzed retrospectively. The documentation took place in Q1/2020. Previous and current treatment and infection data were collected from patients who received a line of therapy for the treatment of CLL or MM starting in 2018 (1st, 2nd, and 3rd or higher line). The time frame was chosen to ensure follow-up for at least 12 months after starting systemic treatment in each line of therapy in order to collect data on susceptibility to infection and possible SID. The methods and analyses used in this study have already been successfully applied and published in comparable studies of the German Cancer Society [20–22].

GLAD was analyzed according to the DGHO-Onkopedia, the recommendations of the German Medical Association, the EMA, and the European consensus proposal for immunoglobulin therapies [14]. The following definitions were used to operationalize the guideline recommendations (GL) for a quantitative analysis: for SID, immunoglobulin substitution (IgRT) is strongly recommended by GL as for patients with an IgG level < 4 g/l (or IgG subclass deficiency) and additionally more than 3 infections or a severe infection (≥ grade 3) and as optional (may be appropriate) if IgG level < 4 g/l and/or 1–3 less severe infections (≤ grade 2). IgRT is not indicated if patients do not fulfil either condition. The initial dose of IgRT should be 0.2–0.4 g/kg bodyweight every 3–4 weeks (± 10% each). The definition of the indication for IgRT by more than 3 occurring infections was chosen according to the cross-sectional guideline of the BÄK from 2014, which was valid at the time of the data collection. The new version of the GL of 2020 (as well as the GL of DGHO and EMA) now only refers to recurrent infections.

For the analysis of GLAD, the following score was developed:

- 2 points for full GLAD.
- 1 point for deviations in initial dose or interval (more than ± 10%) or a late start of IgRT (> 28 days after a severe infection (≥ grade 3).
- 0 points for IgRT without indication (overtherapy) or omitted IgRT despite recommendation (undertherapy). Likewise, 0 points were awarded if both dose and interval deviated from the GL recommendations (e.g., underdosed single dose) or if IgRT was not started until more than 3 months after hypogammaglobulinemia and at least one severe infection.

The vaccination response testing recommended in the guidelines was not analyzed because this test is very rarely used in Germany.

### Statistical methods

The statistical data analysis was performed using R version 4.01 [23]. Descriptive statistics include absolute and relative frequencies for qualitative characteristics. For continuous characteristics, location measures with corresponding measures of dispersion were calculated (median with interquartile range and minimum and maximum).

For the analysis of susceptibility to infection, the time to next infection was examined using the Andersen-Gill model [24], with any infection was counted as event (or any severe infection (grade ≥ 3) for the second model). For effect estimation, hazard ratios were reported with 95% confidence interval in each case. The significance level was set to two-sided ≤ 5% (*p* ≤ 0.05).

For comparisons of interval-scaled variables, the Mann–Whitney *U* test was performed for independent binominal variables. In case of non-binominal independent variables, the Kruskal–Wallis test was used, supplemented by corresponding pairwise comparisons. In order to address the problem of inflation of type I errors by multiple testing, the *p*-values of pairwise comparison were adjusted using the Bonferroni-Holm procedure.

For the primary descriptive analyses, the χ^2^-test according to Pearson was used for categorical variables. The *p*-values of these analyses were adjusted using the Benjamini & Hochberg procedure as far as there was multiple testing for subgroup analysis [25].

The representativeness of the sample was ensured by grounding it in a previously conducted care structure analysis (phase 1). For this purpose, 894 treatment facilities in Germany that potentially treat patients with CLL or MM were contacted. The facilities were asked to provide data on patient volume and key care parameters (clinic/office; certification). Of the centers contacted, 116 clinics and 130 medical practices responded.

### Patients

To avoid a bias in patient selection, the participating centers were asked to consistently document all patients fulfilling the inclusion criteria from 31 December 2018 backwards in time until the specified number of patients to be documented in the respective lines of therapy was reached.

Patients with CLL or MM who had started systemic therapy in 2018 were included. The distribution of patients across therapy lines was carried out according to the previously performed care structure analysis.

Patients who received an allogeneic stem cell transplant and patients in the final phase of their disease with a life expectancy of less than 3 months at the start of therapy were excluded.

### Parameters studied

Patient characteristics, disease parameters, therapies and therapy lines, immunoglobulin monitoring, and the number, type, and severity of infections that occurred were recorded. In addition, infections occurring in the 12 months prior to the start of therapy were also documented to detect any pre-existing tendency to infection. However, the target criterion was number and severity of infections after the start of system therapy. Infection severity was graded according to the CTCAE 5.0 [26].

## Results

### Patient demographics and disease stage and therapy

Data from 1086 patients (CLL 490, MM 596) were documented from 86 centers. The median age at the start of the line of therapy was 73 years (25%, 75% percentiles: 65, 79 years). Four hundred seventy-five (43.7%) of patients were female and 611 (56.3%) were male.

The patients (*n* (%)) were distributed amongst the different disease stages as follows: CLL stage according to Binet: A 169 (34.5%), B 128 (26.1%), C 164 (33.5%), and no information 29 (5.9%); and MM stage according to the (Revised) International Staging System (R-ISS) (if R-ISS was not available, ISS was scored): I 158 (26.5%), II 195 (32.7%), III 178 (29.9%), and no information 65 (10.9%). The Charlson Comorbidity Index (CCI) [27] yielded a median score of 2 points, the 25% and 75% percentiles were 2 and 3 points, and the range was 2 to 9 points for CLL and 2 to 8 points for MM.

Two hundred fifty-three (51.6%) of CLL patients and 286 (48.0%) of MM patients received first-line therapy (Supplementary Fig. 1). The distribution of treatment substances used is shown in Supplementary Table 1.

### Infections

Overall, infections were documented in 410 patients (37.8%), 196 (40.0%) in CLL patients and 214 (35.9%) in MM patients. The number and severity (CTCAE Criteria 5.0) [26] of infections after initiation of therapy are shown in Supplementary Table 3; infections with a severity of grade 3–5 were 28.4% in CLL and 36.9% in MM.

After initiation of the systemic antineoplastic treatment, the number of infections more than doubled (Supplementary Table 3a). However, it is not clear, if all infections were documented, before the patients were treated by a specialist.

Most infections (60.1%) classified by ICD-10 involved the respiratory system; see Supplementary Table 3c.

### Assessment of IgG concentration and IgRT

The examination of IgG levels before and during the analysis period is shown in Supplementary Table 2. IgG levels were determined before therapy in 73.1% of CLL patients and in 89.8% of MM patients. The different lines of therapy showed the following diagnostic rates: CLL: 1st line 75.9%, 2nd line 72.0%, and 3rd and higher line 66.6%; and MM: 1st line 88.8%, 2nd line 91.6%, and 3rd and higher line 89.5%. Immunoglobulin subclasses were determined before therapy in 1.4% of CLL and in 0.4% of MM patients. During the course of therapy, the share of patients whose immunoglobulin subclasses were determined had a maximum of 2.1% and 0.9% of patients. The antibody titer was determined in just one of a total of 87 patients with documented pneumococcal vaccination.

A total of 88.2% of the physicians stated that IgG values were regularly monitored, 42.7% of which were monitored “as standard at every laboratory examination” and 45.5% “regularly but at longer intervals.”

A total of 115 (23.5%) of CLL patients and 86 (14.4%) of MM patients received IgRT. With increasing line of therapy for the underlying disease, the percentage of patients receiving IgRT increased (Table 1).

**Table 1:** Immunoglobulin replacement therapy (IgRT) according to disease

IgRT (CLL)														
			Treatment line											
			1st line				2nd line				3rd + line		Total	
			*N*		%		*N*		%		*N*	%	*N*	%
IgRT	Yes		44		17.4%		40		26.7%		31	35.6%	115	23.5%
	No		202		79.8%		109		72.7%		56	64.4%	367	74.9%
	Unknown/not reported		7		2.8%		1		0.7%		0	0.0%	8	1.6%
	Total		253		100%		150		100%		87	100%	490	100%
IgRT (multiple myeloma)														
		Treatment line												
		1st line				2nd line				3rd + line			Total	
		*N*		%		*N*		%		*N*		%	*N*	%
IgRT	Yes	29		10.1%		22		13.2%		35		24.5%	86	14.4%
	No	250		87.4%		142		85.0%		102		71.3%	494	82.9%
	Unknown/not reported	7		2.4%		3		1.8%		6		4.2%	16	2.7%
	Total	286		100%		167		100%		143		100%	596	100%

This table shows whether IgRT has taken place, regardless of dose and interval, as well as start time

### Guideline adherence (GLAD score)

**Fig. 1:**
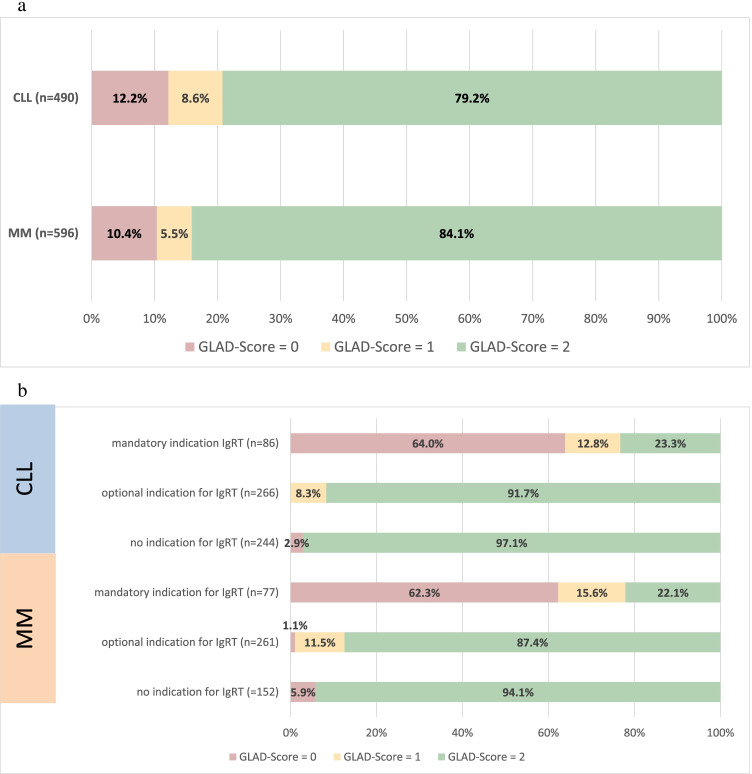
**a** Guideline adherence score (GLAD score); CLL, chronic lymphocytic leukemia; MM, multiple myeloma. **b** GLAD scores in patients with hypogammaglobulinemia and indication for IgRT

Analysis according to the GLAD score showed good GL adherence (score 2) in 388 (79.2%) of the CLL patients and in 501 (84.1%) of the MM patients (Fig. 1a). The deviations (scores 0 and 1) were predominantly undertherapy or too long intervals between IgG infusions.

Most patients with GLAD score 1 received a downward dose deviation (80%, *n* = 60), so that IgRT dose was lower than 0.18 g/kg bodyweight (− 10% tolerance from recommended minimum dose of 0.2 g/kg bodyweight). The remaining 20% of patients with GLAD score 1 resulted from interval extensions (12%; *n* = 9) and/or delayed start of IgRT > 28 days (~ 7%, *n* = 5) and dose upward deviation (4%, *n* = 3). In the patients with downward deviation, there were also patients for whom IgRT was started too late (therefore the total is > 100%). 78.7% (*n* = 96) of the patients with GLAD score 0 received no IgRT despite indication (undertherapy), in 8.2% (*n* = 10), the IgRT started more than 3 months after severe infection, and in 13.1% (*n* = 16) of patients, an IgRT was initiated without indication (overtherapy).

Considering GLAD only for patients with a mandatory indication, only 23.3% of the 86 CLL patients and 22.1% of the 77 MM patients achieved a GLAD score of 2 (Fig. 1b).

The differences between non-certified and certified centers (certified by German Cancer Society, German Society of Hematology and Medical Oncology or Comprehensive Cancer Centers) in GLAD (80.4% vs. 84.5%) are not significant across both indications: odds ratio (OR) 1.33 (95% CI 0.95–1.85; *p* = 0.140). In the subgroup analysis of the two indications, the CLL subgroup shows a difference in favor of certified centers, which is also not significant (after FDR adjustment) (76.4% vs. 84.5%): OR 1.69 (95% CI 1.03–2.76; *p* = 0.105). In contrast, no differences were observed in MM (83.8% vs. 84.5%): OR 1.05 (95% CI 0.67–1.67; *p* = 0.833). Differences in the type of care (hospitals vs. office-based hematologists) could not be determined either (82.6% vs. 81.6%): OR 0.93 (95% CI 0.65–1.34; *p* = 0.997).

### Guideline adherence and susceptibility to infections

**Fig. 2:**
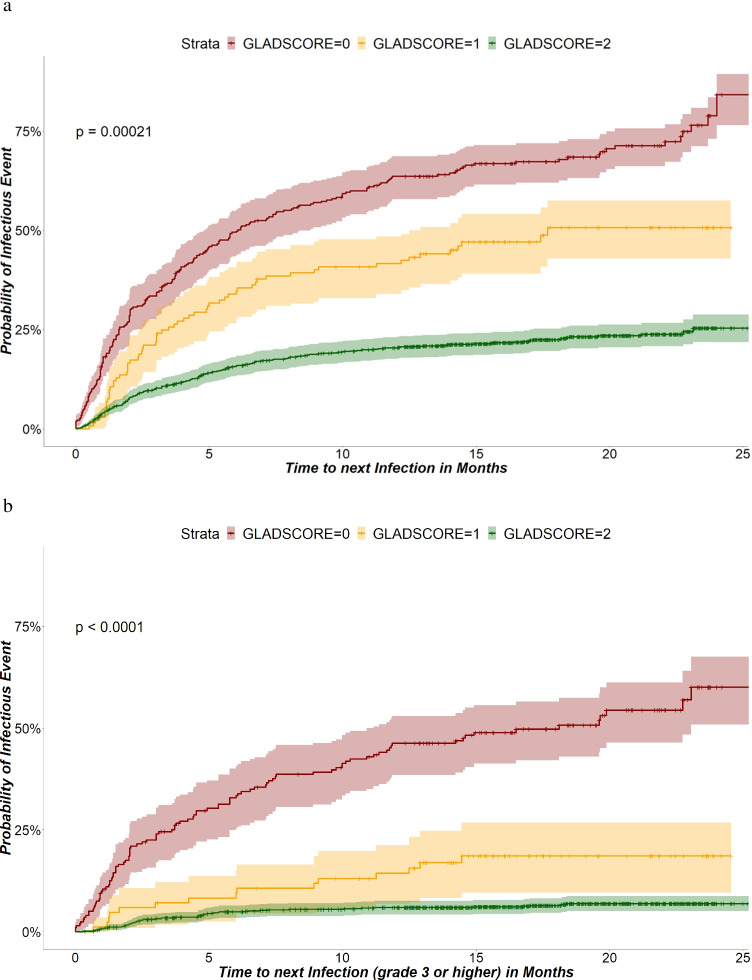
**a** GLAD score and time to next infectious event—all infections. **b** GLAD score and time to next infectious event—severe infections (grades ≥ 3)

The likelihood of infection was significantly lower at a GLAD score of 2 or 1 than at 0 (Fig. 2a and b).

The GLAD score correlated significantly with the relative likelihood of infection: compared to full GLAD (GLAD score = 2: reference), the hazard ratio (HR) for infection at a GLAD score 1 was 2.52 (95% CI 1.98–3.21; *p* < 0.001). For a GLAD score 0, the HR was 4.49 (95% CI 3.72–5.42; *p* < 0.001).

The relative likelihood of severe infections (grade ≥ 3) also correlated with GLAD. The hazard ratio (HR) was 2.96 (95% CI 1.67–5.25; *p* < 0.001) for GLAD score 1 and 10.64 (95% CI 7.54–15.00; *p* < 0.001) for GLAD score 0.

### Guideline adherence and severity of infections

Besides the fact that significantly more infections occurred with a lower GLAD score, the incident infections were also more severe in patients with a GLAD score 0 than in those with a score 1 or 2 (see Table 2) (*p* < 0.001), although the differences between GLAD score 2 and GLAD score 1 were not clear. Notwithstanding that the infections occurring in GLAD score 2 and 1 were just as often grade ≥ 3 (23.6% vs. 23.8%), infections in GLAD score 1 were more often fatal (2.4% vs. 7.9%).

**Table 2:** GLAD score and severity of infections [26]

GLAD score								
	0		1		2		Total	
	*N*	%	*N*	%	*N*	%	*N*	%
Grade 1	31	14.0%	17	27.0%	63	25.2%	111	20.8%
Grade 2	89	40.3%	31	49.2%	128	51.2%	248	46.4%
Grade 3	75	33.9%	10	15.9%	52	20.8%	137	25.7%
Grade 4	4	1.8%	0	0.0%	1	0.4%	5	0.9%
Grade 5	22	10.0%	5	7.9%	6	2.4%	33	6.2%
Total	221	100%	63	100%	250	100%	534	100%

Grade 1: no intervention indicated

Grade 2: oral intervention indicated; e.g., antibiotic, antifungal, or antiviral

Grade 3: hospitalization and/or IV antibiotic, antifungal, or antiviral intervention indicated

Grade 4: life-threatening consequences; urgent intervention indicated

Grade 5: death

At a lower GLAD score, infections were significantly more severe, *p* < 0.001 (Kruskal–Wallis test). Pairwise comparison shows that at a GLAD score of 0, infections were significantly more severe than at a GLAD score of 1 (*p* = 0.003) and of 2 (*p* < 0.001), whereas the pairwise comparison of GLAD score of 1 and 2 was not significant (*p* = 1)

**Fig. 3:**
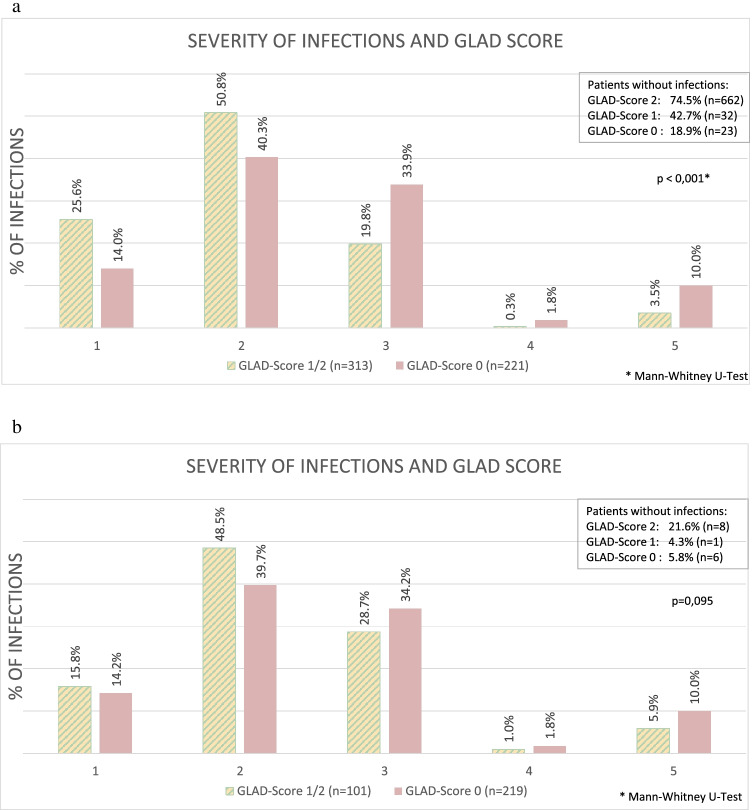
GLAD score 1/2 vs 0, in case of mandatory indication and severity of infections. Severity of infections according to CTCAE [4]; grade 1: asymptomatic or mild symptoms; grade 2: oral intervention indicated (e.g., antibiotic, antifungal, or antiviral); grade 3: IV antibiotic, antifungal, or antiviral intervention indicated; invasive intervention indicated; grade 4: life-threatening consequences; urgent intervention indicated; Grade 5: death. **a** All patients, severity of infections at GLAD score 0 compared to pooled GLAD scores 1 and 2. **b** Patients with indication for IgRT according to guidelines, severity of infections at GLAD score 0 compared to the combined GLAD scores 1and 2

Since the groups GLAD scores 1 and 2 differed not in terms of severity of infections in the pairwise comparison (*p* = 1.000), the group GLAD score 0, however, showed significantly different results (GLAD score 1 vs. 0: *p* = 0.003; 2 vs. 0: *p* < 0.001). GLAD scores 1 and 2 were combined and compared with GLAD score 0 (*p* < 0.001); see Fig. 3a.

When considering only patients with the mandatory indication for IgRT, the severity of infection was higher for GLAD score 0 (*p* = 0.235) as well. Summarizing the severity of infection by GLAD score (GLAD score 1/2) and analyzing only patients with a mandatory indication, more severe infections occurred with a GLAD score of 0 (46.1% vs. 35.6%), but again the significance level was not reached (*p* = 0.095) (Fig. 3b). However, it is striking that the proportion of fatal infections in this subgroup was also higher with a GLAD score of 0 (10.0%, *n* = 22) than with a GLAD score of 1/2 (5.9%, *n* = 6), even if the number of fatal infections was too small to measure a statistically significant difference.

### Risk factors for infectious events

**Fig. 4:**
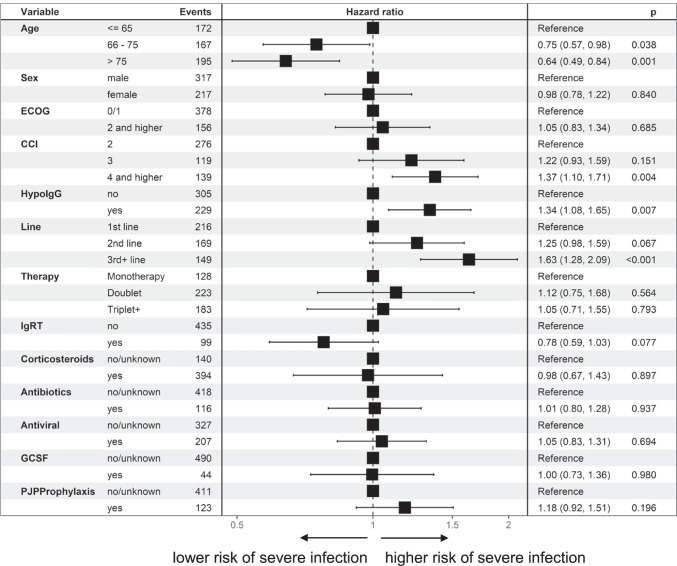
Risk factors for an increased incidence of infections, for different risk factors all infections in all patients, (multivariable Anderson-Gill model)

Risk factors for increased susceptibility to infection (all infections) in all patients in the multivariable AG model were an increased Charlson Comorbidity Index (CCI, HR 1.37 (95% CI 1.10–1.71; *p* = 0.004), existing hypogammaglobulinemia below 4 g/l (HR 1.34 (95% CI 1.08–1.65); *p* = 0.0047) and advanced line of therapy (3rd + line) (HR 1.63 (95% CI 1.28–2.09); *p* < 0.001) (Fig. 4). Older patients over 75 years of age had a lower risk here (HR 0.64 (95% CI 0.49–0.84); *p* = 0.002). For IgRT, there was a trend towards a lower risk of infection under IgRT than without/before IgRT, but this did not reach the significance level (HR 0.78 (95% CI 0.59–1.03); *p* = 0.077).

**Fig. 5:**
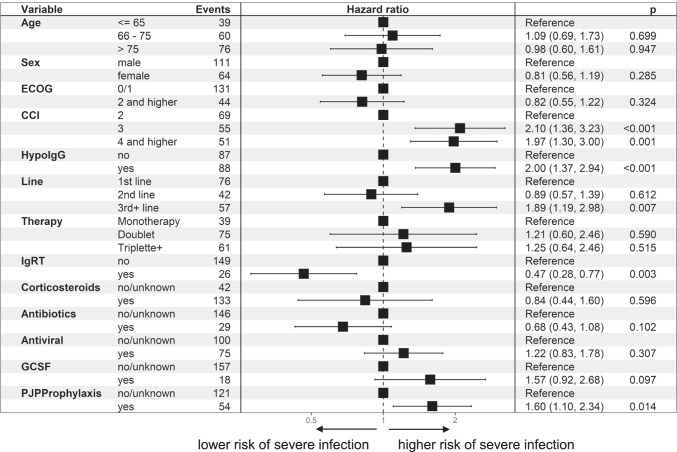
Risk factors for an increased incidence of infections, for different risk factors, all patients, hazard ratio (hazard of severe infection [grade=3]) for different risk factors (multivariable Anderson-Gill model)

**Fig. 6:**
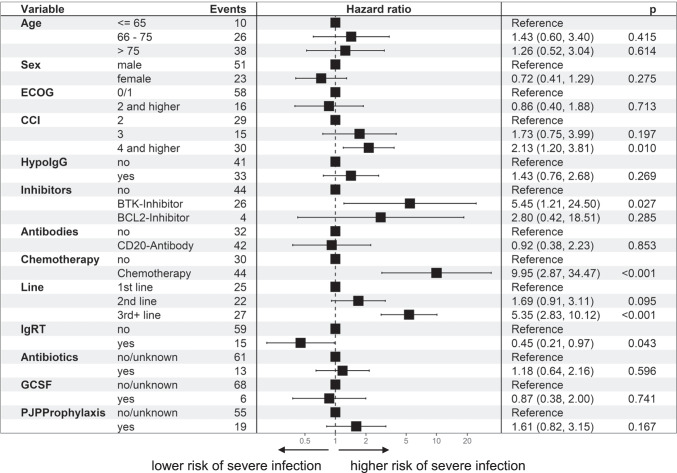
Risk factors for an increased incidence of infections, CLL, hazard ratio (hazard of severe infection [grade=3]) for different risk factors (multivariable Anderson-Gill model)

With regard to severe infections (grade ≥ 3), the risk factors were essentially confirmed and, with the exception of age, emerge even more clearly (Figs. 5, 6 and7): CCI of 4 or more HR 1.97 (95% CI 1.30–3.00; *p* = 0.001) compared to CCI of 2, which is due to the underlying malignancy; and hypogammaglobulinemia HR 2.00 (95% CI 1.37–2.94; *p* < 0.001), later line of therapy HR 1.89 (95% CI 1.19–2.89; *p* = 0.007). IgRT was associated with a lower risk, HR 0.47 (95% CI 0.28–0.77; *p* = 0.003). There was no difference in more severe infections for older age: HR 0.98 (95% CI 0.60–1.61; *p* = 0.947). Significantly, however, patients on *Pneumocystis jirovecii* prophylaxis were also more prone to severe infections HR 1.60 (95% CI 1.10–2.34; *p* < 0.014).

**Fig. 7:**
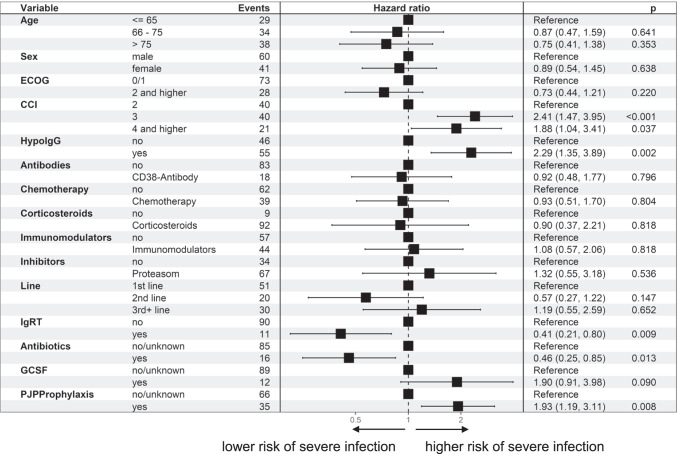
Risk factors for an increased incidence of infections, Multiple myeloma, hazard ratio (hazard of severe infection [grade=3]) for different risk factors (multivariable Anderson-Gill model)]

In the subgroup of CLL, patients treated with BTK inhibitors (HR 5.54 (95% CI 1.21–24.49; *p* = 0.027)) and chemotherapy (HR 5.54 (95% CI 1.21–24.49) *p* = 0.027) had a higher risk for severe infections. Treatment with CD20 antibodies did not make a difference in susceptibility to severe infections (HR 0.92 (95% CI 0.38–2.23); *p* = 0.853). A higher CCI (≥ 4) as well as a later line of therapy (3^rd^ +) could be confirmed as risk factors: CCI: HR 2.12 (95% CI 1.20–3.81, *p* = 0.010); and 3^rd^ + : HR 5.35 (95% CI 2–83–10.12, *p* < 0,001), whereas an existing hypogammaglobulinemia was not: HR 1.43 (95% CI 0.76–2.68; *p* = 0.269).

In MM patients, an antibiotic prophylaxis correlated with a lower risk of infection: HR 0.43(95% CI 0.25–0.85; *p* = 0.013). A different risk for different treatment options could not be measured. The higher risk for severe infections in patients with a higher CCI as well as in patients with hypogammaglobulinemia was confirmed in the MM subgroup, but later line of therapy does not correlate with a higher risk (Fig. 7). There seems to be a trend (not statistically significant) that patients treated with G-CSF prophylaxis had a higher risk of severe infections: HR 1.90 (95% CI 0.91–3.98; *p* = 0.090), but it has to be considered that the proportion of patients with G-CSF prophylaxis is relatively low (9.2% *n* = 55) and only 50.9% (*n* = 28) of them were treated with a primary G-CSF prophylaxis.

## Discussion

The number of patients treated adherent to guidelines (GLAD) to immunoglobulin substitution in Germany is relatively high, with 79.2% in CLL and 84.1% in MM. However, a reason for high GLAD may be that IgRT is only recommended for a subgroup of patients, in whom IgRT is essentially neglected: GLAD is poor in patients with mandatory indication for IgRT, with a GLAD score of 0 in 64.0% of CLL and 62.3% of MM patients.

There is a significant correlation between the level of GLAD and the cumulative incidence of infection over the median study observation period of 18.2 months. Even with less than optimal GLAD, the cumulative rate of infections was lower than in patients without high GLAD. This supports the recent European expert conference on recommendations for the diagnosis and treatment of antibody deficiency with IgRT in patients with malignant hematologic diseases [28]. There may be various reasons for non-compliance with the guidelines, but it should not be the cost of the immunoglobulins, because drugs used in accordance with the approval are paid for by the health insurance funds in Germany.

Several independent risk factors for severe infections could be identified, such as an elevated Charlson Comorbidity Index (CCI) of 3 or more, hypogammaglobulinemia, and third-line or higher therapy. IgRT, on the other hand, had a significantly protective effect. If these factors are analyzed separately for CLL and MM, these effects remain in CLL, whereas in MM, line of therapy no longer plays a role, but antibiotic prophylaxis is beneficial. Prophylaxis against *Pneumocystis jirovecii* correlated with a higher risk, possibly because these are patients who are at higher risk of infection overall, so appropriate prophylaxis was given. Antibiotic prophylaxis with levofloxacin in newly diagnosed MM during the first 12 weeks of therapy significantly reduces infections [29], which is in line with the results of our study. At the first glance, G-CSF to prevent neutropenia seems to correlate with a higher risk of severe infections, even if this is not statistically significant. But it has to be considered that patients with G-CSF prophylaxis are treated with more aggressive regimens, for which G-CSF prophylaxis is appropriate [2, 30, 31].

In the present multivariable analysis, patients on BTK inhibitor therapy had a significantly increased risk of infection. In contrast to other studies [32], no increased risk for CLL patients treated with CD20 antibodies such as rituximab and subsequent hypogammaglobulinemia could be measured in this study. However, this may also be due to the fact that about just under two-thirds of the patients not presently treated with CD20 antibodies had already received CD20 antibodies in a previous therapy. It should also be considered that B-cell depletion persists long after the end of treatment, especially in combination with fludarabine [33, 34]. One study showed that IgRT significantly reduced the risk of infection in patients with hypogammaglobulinemia [35].

A study with a smaller number of patients with newly diagnosed CLL [36] did not find a significant effect of hypogammaglobulinemia on infections. However, our study showed a significantly lower rate of severe infections with IgRT in patients overall, compared to patients before or without IgRT. Patients with an indication for IgRT and poor GL adherence according to GLAD had more and more severe infections.

In MM, antibody deficiency and in particular the deficit of specific antibodies leads to a higher rate of infection [37]. Our analysis confirms the increased risk of severe infection in hypogammaglobulinemia and the positive effect of IgRT. In patients with CLL, it is known that higher comorbidity is correlated with poorer survival. This is also true for the Charlson Comorbidity Index (CCI) [38] used here. In MM, patient comorbidity correlates with prognosis [39–41] as well. We were able to show that this is also true for the risk of severe infection. Older age was not a risk factor for severe infections in neither CLL nor MM. Interestingly, age over 75 years was a favorable risk factor when all infections were considered. This may be explained by an age-adapted, i.e., less aggressive therapy of the primary disease, resulting in fewer therapy-associated complications and infections.

The limitations of the retrospective study are that the information in the patient records cannot be verified and that diagnosis and monitoring of the patients are not specified. In the case of infections, the classification was missing for a large number of patients. The course of infection depends not only on prophylaxis but also on the corresponding therapy, on which we have no data.

Even if one can criticize the guidelines for IgRT in detail, because the evidence refers to older studies whose therapy of CLL or MM is now partly considered outdated, and because infections must already have occurred for the indication to be made, they are nevertheless well suited for everyday clinical use. Patients for whom IgRT is clinically important, especially to avoid severe infections, can be identified. The IgRT guidelines against which adherence was measured are international. Therefore, the correlation of the GLAD score with infections and the risk factors for infections are generalizable. The implementation of the GL should be improved both in diagnostics and IgRT. Especially in the group with mandatory indication, i.e., in the risk group of patients who have a clinical infection problem, an improvement in IgRT uptake as per guidelines will lead to a clinical benefit with infections. Nevertheless, prospectively designed studies would be useful to develop and verify practicable algorithms or risk scores in clinical practice in order to better predict the probability of infection and thus the indication for IgRT in the light of current therapies.

## Supplementary Information

Below is the link to the electronic supplementary material.

Supplementary file1 (PDF 754 KB)

## Data Availability

The funding source did not have any access to the data and was not involved in data analysis or manuscript writing. The authors confirm that they have full control of all primary data and agree to allow the journal to review their data if requested.

## Declarations

### Standard of reporting

The guidelines for Observational Studies in Epidemiology (STROBE) were taken into account as far as applicable [42].
