# Supplementary material for: Immunoglobulin substitution in patients with secondary antibody deficiency in chronic lymphocytic leukemia and multiple myeloma: a representative analysis of guideline adherence and infections
Source: Support Care Cancer. 2022 Mar 7;30(6):5187–200. doi: 10.1007/s00520-022-06920-y (PMC9046374; doi:10.1007/s00520-022-06920-y)
Supplement: Supplementary file 1 — Supplementary file1 (PDF 754 KB) [file 520_2022_6920_MOESM1_ESM.pdf]

Flow Diagram, number (n) of patients

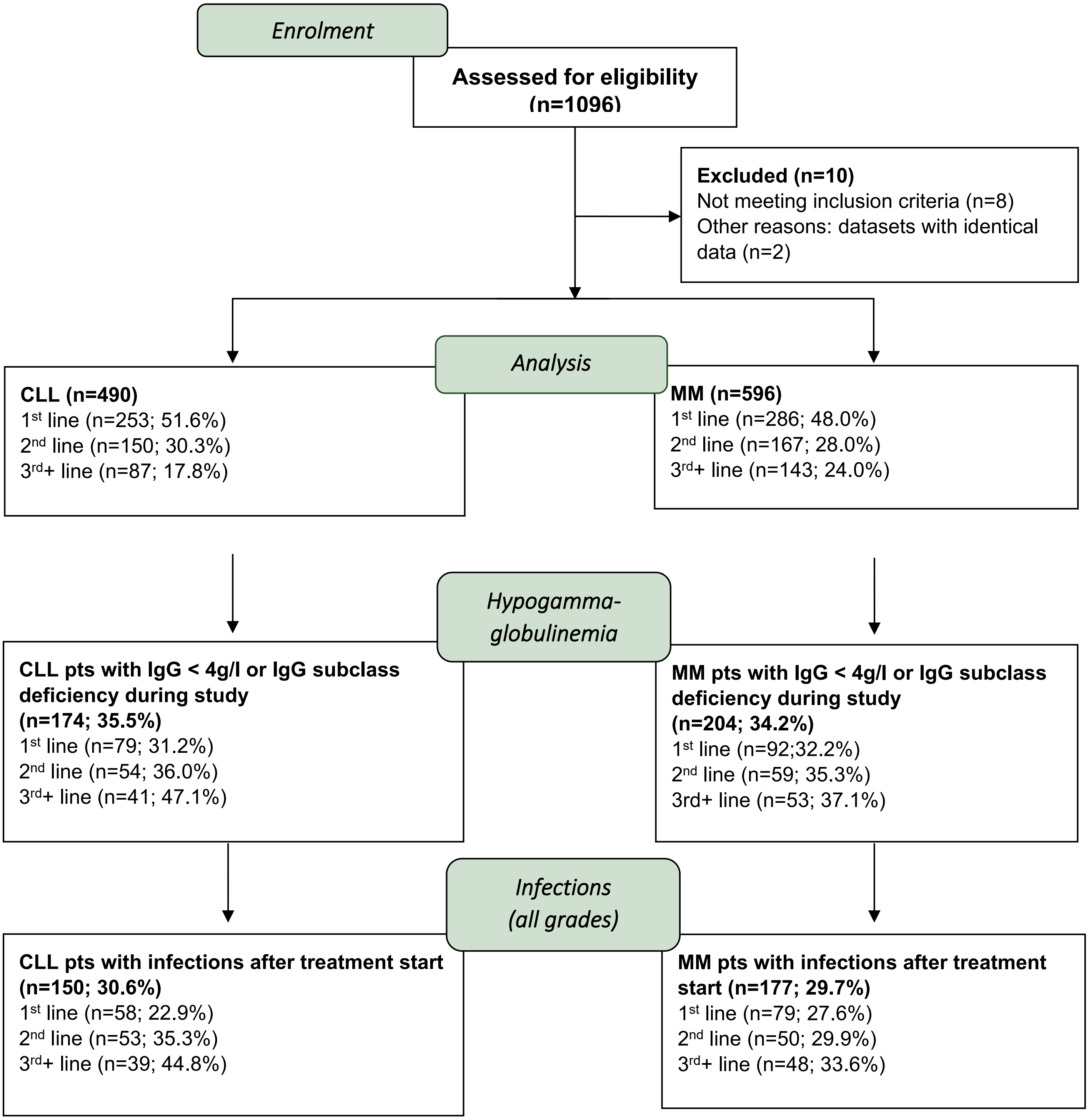

Supplementary Figure 1: Consort-Chart of the included patients, CLL - chronic lymphocytic leukemia, MM – multiple myeloma, pts - patients; line - line of therapy

Supplementary Table 1a: Systemic Treatment Substances - CLL

		Treatment line					
		1st line		2nd line		3rd+ line	
		N	%	N	%	N	%
**Systemic treatment – substances**	**Bendamustine**	119	47.0%	44	29.3%	10	11.5%
	**Chlorambucil**	39	15.4%	12	8.0%	5	5.7%
	**Cyclophosphamide**	32	12.6%	5	3.3%	2	2.3%
	**Doxorubicin**	0	0.0%	2	1.3%	1	1.1%
	**Fludarabine**	36	14.2%	6	4.0%	1	1.1%
	**Ibrutinib**	38	15.0%	62	41.3%	41	47.1%
	**Idelalisib**	3	1.2%	18	12.0%	4	4.6%
	**Obinutuzumab**	31	12.3%	6	4.0%	7	8.0%
	**Ofatumumab**	0	0.0%	1	0.7%	2	2.3%
	**Pixantrone**	0	0.0%	1	0.7%	0	0.0%
	**Prednisolone**	1	0.4%	2	1.3%	3	3.4%
	**Rituximab**	164	64.8%	70	46.7%	12	13.8%
	**Tirabrutinib**	0	0.0%	2	1.3%	1	1.1%
	**Venetoclax**	17	6.7%	3	2.0%	19	21.8%
	**Vincristine**	0	0.0%	2	1.3%	1	1.1%

Supplementary Table 1b: Systemic Treatment Substances - MM

		Treatment line					
		1st line		2nd line		3rd+ line	
		N	%	N	%	N	%
**Systemic treatment – substances**	**Azacitidine**	1	0.3%	0	0.0%	0	0.0%
	**Bendamustine**	10	3.5%	0	0.0%	3	2.1%
	**Bortezomib**	227	79.4%	49	29.3%	26	18.2%
	**Carfilzomib**	2	0.7%	19	11.4%	29	20.3%
	**Cyclophosphamide**	116	40.6%	11	6.6%	8	5.6%
	**Daratumumab**	9	3.1%	35	21.0%	49	34.3%
	**Dexamethasone**	238	83.2%	131	78.4%	107	74.8%
	**Doxorubicin**	1	0.3%	3	1.8%	2	1.4%
	**Elotuzumab**	1	0.3%	16	9.6%	11	7.7%
	**Ixazomib**	1	0.3%	2	1.2%	3	2.1%
	**Lenalidomide**	59	20.6%	98	58.7%	65	45.5%
	**Melphalan**	42	14.7%	13	7.8%	3	2.1%
	**Panobinostat**	0	0.0%	0	0.0%	1	0.7%
	**Pomalidomide**	0	0.0%	4	2.4%	10	7.0%
	**Prednisolone**	41	14.3%	12	7.2%	4	2.8%
	**Rituximab**	0	0.0%	0	0.0%	1	0.7%
	**Thalidomide**	0	0.0%	2	1.2%	0	0.0%
	**Vincristine**	0	0.0%	0	0.0%	1	0.7%

Supplementary Table 2: Ig G serum value diagnostics before and after start of systemic treatment

		Disease					
		CLL			Multiple Myeloma		
		N	% (all pts)	% (pts with valid n)	N	% (all pts)	% (pts with valid n)
**IgG level (before start of systemic treatment)**	**not determined**	132	26.9%	0.0%	61	10.2%	0.0%
	**<4g/l**	85	17.3%	23.7%	94	15.8%	17.6%
	**4 - <7g/l**	120	24.5%	33.5%	91	15.3%	17.0%
	**7 - 16g/l**	141	28.8%	39.4%	94	15.8%	17.6%
	**>16g/l**	12	2.4%	3.4%	256	43.0%	47.9%
	**deceased**	0	0.0%	0.0%	0	0.0%	0.0%
	**Total**	490	100%	100%	596	100%	100%
**IgG level (Q1 after start of systemic treatment)**	**not determined**	270	55.1%	0.0%	95	15.9%	0.0%
	**<4g/l**	44	9.0%	20.4%	98	16.4%	19.9%
	**4 - <7g/l**	112	22.9%	51.9%	109	18.3%	22.1%
	**7 - 16g/l**	53	10.8%	24.5%	177	29.7%	35.9%
	**>16g/l**	7	1.4%	3.2%	109	18.3%	22.1%
	**deceased**	4	0.8%	0.0%	8	1.3%	0.0%
	**Total**	490	100%	100%	596	100%	100%
**IgG level (Q2 after start of systemic treatment)**	**not determined**	240	49.0%	0.0%	106	17.8%	0.0%
	**<4g/l**	52	10.6%	21.8%	81	13.6%	17.8%
	**4 - <7g/l**	128	26.1%	53.6%	138	23.2%	30.4%
	**7 - 16g/l**	56	11.4%	23.4%	163	27.3%	35.9%
	**>16g/l**	3	0.6%	1.3%	72	12.1%	15.9%
	**deceased**	11	2.2%	0.0%	36	6.0%	0.0%
	**Total**	490	100%	100%	596	100%	100%
**IgG level (Q3 after start of systemic treatment)**	**not determined**	243	49.6%	0.0%	99	16.6%	0.0%
	**<4g/l**	39	8.0%	17.3%	70	11.7%	15.9%
	**4 - <7g/l**	107	21.8%	47.3%	119	20.0%	27.1%
	**7 - 16g/l**	75	15.3%	33.2%	190	31.9%	43.3%
	**>16g/l**	5	1.0%	2.2%	60	10.1%	13.7%
	**deceased**	21	4.3%	0.0%	58	9.7%	0.0%
	**Total**	490	100%	100%	596	100%	100%
**IgG level (Q4 after start of systemic treatment)**	**not determined**	241	49.2%	0.0%	114	19.1%	0.0%
	**<4g/l**	35	7.1%	16.0%	57	9.6%	13.9%
	**4 - <7g/l**	115	23.5%	52.5%	105	17.6%	25.6%
	**7 - 16g/l**	65	13.3%	29.7%	195	32.7%	47.6%
	**>16g/l**	4	0.8%	1.8%	53	8.9%	12.9%
	**deceased**	30	6.1%	0.0%	72	12.1%	0.0%
	**Total**	490	100%	100%	596	100%	100%

Documented is the lowest measured IgG value within the respective quarter after the start of systemic treatment.

Supplementary Table 3: Incidence and Severity of Infections

Supplementary Table S3a: Number of infections before and after start of systemic treatment

	Disease					
	CLL		Multiple Myeloma		Total	
	N	%	N	%	N	%
**Infections before start of systemic treatment**	121	31.8%	96	25.9%	217	28.9%
**Infections after start of systemic treatment**	260	68.2%	274	74.1%	534	71.1%
**Total**	381	100.0%	370	100.0%	751	100.0%

The number refers to documented infections. so multiple responses are included (each patient may have more than one infection).

Supplementary Table 3b: Severity of infections after start of treatment according to CTCAE 5.0 criteria [4]

	Disease					
	CLL		Multiple Myeloma		Total	
	N	%	N	%	N	%
**Grade 1**	52	20.0%	59	21.5%	111	20.8%
**Grade 2**	134	51.5%	114	41.6%	248	46.4%
**Grade 3**	62	23.8%	75	27.4%	137	25.7%
**Grade 4**	0	0.0%	5	1.8%	5	0.9%
**Grade 5**	12	4.6%	21	7.7%	33	6.2%
**Total**	260	100.0%	274	100.0%	534	100.0%

Grade 1: no intervention indicated
Grade 2: oral intervention indicated; e.g., antibiotic, antifungal, or antiviral
Grade 3: hospitalization and/or IV antibiotic, antifungal, or antiviral intervention indicated
Grade 4: life-threatening consequences; urgent intervention indicated
Grade 5: death

Supplementary Table 3c: Type of infection after start of systemic treatment according to ICD-10

		CLL		Disease Multiple Myeloma		Total	
		N	%	N	%	N	%
ICD-10-Chapter	I Certain infectious and parasitic diseases	45	17.3%	65	23.7%	110	20.6%
	VI Diseases of the nervous system	1	0.4%	0	0.0%	1	0.2%
	VII Diseases of the eye and adnexa	1	0.4%	3	1.1%	4	0.7%
	IX Diseases of the circulatory system	2	0.8%	1	0.4%	3	0.6%
	X Diseases of the respiratory system	166	63.8%	155	56.6%	321	60.1%
	XI Diseases of the digestive system	5	1.9%	3	1.1%	8	1.5%
	XII Diseases of the skin and subcutaneous tissue	10	3.8%	5	1.8%	15	2.8%
	XIV Diseases of the genitourinary system	16	6.2%	20	7.3%	36	6.7%
	XVIII Symptoms. signs and abnormal clinical and laboratory findings, not elsewhere classified	14	5.4%	22	8.0%	36	6.7%
	Total	260	100.0%	274	100.0%	534	100.0%
